# The Phloem-Sap Feeding Mealybug (*Ferrisia virgata*) Carries ‘*Candidatus* Liberibacter asiaticus’ Populations That Do Not Cause Disease in Host Plants

**DOI:** 10.1371/journal.pone.0085503

**Published:** 2014-01-20

**Authors:** Marco Pitino, Michele T. Hoffman, Lijuan Zhou, David G. Hall, Ian C. Stocks, Yongping Duan

**Affiliations:** 1 United States Horticultural Research Laboratory, United States Department of Agriculture-Agriculture Research Service, Fort Pierce, Florida, United States of America; 2 University of Florida, Institute of Food and Agricultural Sciences-Indian River Research and Education Center, Fort Pierce, Florida, United States of America; 3 Florida Department of Agriculture and Consumer Services-Drug Policy Institute, Gainesville, Florida, United States of America; Key Laboratory of Horticultural Plant Biology (MOE), China

## Abstract

*‘Candidatus* Liberibacter asiaticus’ (Las) is the primary causal agent of huanglongbing (HLB), the most devastating disease of citrus worldwide. There are three known insect vectors of the HLB-associated bacteria, and all are members of the Hemiptera: *Diaphorina citri* (Psyllidae), *Trioza erytreae* (Triozidae), and *Cacopsylla* (*Psylla*) *citrisuga* (Psyllidae). In this study, we found that another hemipteran, the striped mealybug *Ferrisia virgata* (Cockerell) (Hemiptera: Pseudococcidae), was able to acquire and retain Las bacteria. The bacterial titers were positively correlated with the feeding acquisition time on Las-infected leaf discs, with a two-weeks feeding period resulting in Ct values ranging from 23.1 to 36.1 (8.24×10^7^ to 1.07×10^4^ Las cells per mealybug). We further discovered that the prophage/phage populations of Las in the mealybugs were different from those of Las in psyllids based on Las prophage-specific molecular markers: infected psyllids harbored the Las populations with prophage/phage FP1 and FP2, while infected mealybugs carried the Las populations with the iFP3 being the dominant prophage/phage. As in the psyllids, Las bacteria were shown to move through the insect gut wall to the salivary glands after being ingested by the mealybug based on a time-course quantitative polymerase chain reaction (qPCR) assay of the dissected digestive systems. However, Las populations transmitted by the mealybugs did not cause disease in host plants. This is the first evidence of genetic difference among Las populations harbored by different insect vectors and difference among Las populations with respect to whether or not they cause disease in host plants.

## Introduction

*‘Candidatus* Liberibacter asiaticus’ (Las) is the primary causal agent of citrus huanglongbing (HLB), the most devastating disease of citrus worldwide [1]. The HLB bacteria, comprising three species of ‘*Ca.* Liberibacter’, are gram-negative, alpha-proteobacteria that reside within sieve tube cells of infected plants [1], [2], [3], [4], [5]. The disease symptoms include yellow shoots, vein yellowing, asymmetric blotchy-mottled leaves, leaf curl with vein corking, tree decline and ultimately plant death [1], [6].

Three psyllid species are known insect vectors of HLB bacteria: *Diaphorina citri* (Hemiptera: Liviidae), *Cacopsylla citrisuga* (Hemiptera: Psyllidae), and *Trioza erytreae* (Hemiptera: Triozidae) [7]. Periwinkle (*Catharanthus roseus*) is an experimental host of the HLB bacteria and can be infected via parasitic dodder (*Cuscuta campestri*) or by grafting from infected periwinkle to healthy plants [8], [9]. Periwinkle plants become infected with Las quickly and display disease symptoms characteristic of HLB in citrus [1], [10]. Interestingly, the pathogen multiplies more rapidly in periwinkle plants compared to citrus, in which Las maintains relatively low titers [11].

Vector transmission processes are often diverse; some are simple mechanical transmission, where the pathogen is transmitted to plant via the stylet tips during feeding, while others, such as HLB bacteria, must circulate through the body of the vector, cross the intestinal epithelium and salivary gland barriers and finally transmit into the plants [12]. In the order ‘Homoptera’, the midgut and salivary glands of the digestive system are organs of specificity in the transmission pathway [13]. In psyllids, HLB bacteria must first cross intestinal epithelial and salivary gland barriers and circulate in the hemolymph before finally being transmitted to the plants [14]. The gut wall and salivary glands are the most important barriers of persistently and propagatively transmitted plant pathogens [15], [16], [17], [18], [19].

Previously, we reported the detection of Las bacteria from 63% of striped mealybugs, *Ferrisia virgata* (Cockerell) (Hemiptera: Pseudococcidae) collected from Las-infected periwinkle plants [20]. This mealybug is a phloem-sap feeding insect with a broad host range of more than 150 genera in 68 families including citrus and periwinkle [21]. Mealybugs are important plant pests because they can cause economic damage to plants by direct feeding damage, introducing salivary secretions that are toxic to plants, excreting honeydew onto leaves, which promotes the development of photosynthesis-blocking sooty mold, and some species of mealybugs transmit plant pathogens [22]. Mealybugs are similar to other phloem-feeding Hemiptera such as whiteflies and psyllids with respect to how they use their stylets to feed [22], [23].

The dynamics of Las populations in host plants and insects are mediated by their prophage/phage activities [24]. A total of nine types of prophages/phages have been identified in Florida Las isolates. Types A and B share highly conserved sequences localized within the two prophages, FP1 and FP2, respectively, while the type D localizes to iFP3 (the recombinant of FP1 and FP2). Only types A and B are abundant in Las-infected psyllids, while type D is undetectable in almost all infected psyllids. The presence of iFP3 is also associated with blotchy mottle of leaves, a phenotype of disease attenuation [24].

In this study, we investigated acquisition and transmission efficiencies of Las by the striped mealybugs, and used Las specific molecular markers to differentiate the different Las populations present in *F. virgata* and *D. citri*. Results indicate both insect hosts are able to acquire and carry different populations of Las bacteria, but only psyllids are able to transmit the Las populations that cause disease in host plants.

## Materials and Methods

### Insects

The insects for the study were maintained in separate cages located in a greenhouse at the USDA-ARS facility in Fort Pierce, FL. *Ferrisia virgata* were reared on healthy periwinkle, *Catharanthus roseus,* grown from seed in our greenhouses. *Diaphorina citri* were reared on healthy citrus following the general procedures outlined by Skelley and Hoy [25].

### Leaf Disc Bioassay

Leaf discs were cut from leaves of Las-infected periwinkle and Las-infected old or young citrus leaves using an 11 mm diameter borer and procedures as described in Pitino et al. [26]. They were then individually placed in single wells of a 24-well plate on top of 2 ml of solidified 1% distilled water agar (DWA). One third-instar nymph reared on a non-infected plant was placed onto each leaf disc for a total of 24 leaf discs per plate. The plates were individually sealed with mesh and placed upside down, and the mealybugs were left on the leaf disc for either 1 or 2 weeks. After the first week, 12 leaf discs and their corresponding mealybugs were analyzed by quantitative polymerase chain reaction (qPCR) using 16S rDNA-based primers and a TaqMan-based probe [27]. The other 12 mealybugs were transferred to new healthy periwinkle or citrus leaf discs for transmission assays. The same procedure was repeated with two new cohorts, one per week, of one third-instar per disc (n = 24). Negative control mealybugs on healthy leaf discs were left feeding for 2 weeks and then analyzed by qPCR. The experiments were repeated in triplicate.

### Whole Plant Assay

Transmission efficiency studies were conducted using non-infected periwinkle plants and clip cages. Ten Las-infected mealybug adults, previously reared on infected periwinkle leaves for 2 weeks, were placed inside clip cages attached to the uppermost leaf on the test plant. We used six periwinkle plants with one clip cage for the first plant and added one additional clip cage per additional plant; thus, the number of infected mealybugs began with 10 on the first plant and ended with 60 on the last plant with increment of 10 insects per plant. New mealybug nymphs were removed from each cage every 4 days. Adult mealybugs were left on the plants for 4 weeks. After the second, third and fourth week, n = 10 mealybugs were collected randomly from clip cages for qPCR analysis. Plants were grown for an additional twelve months in insect-proof greenhouse, and were assessed for visual symptoms of infection and analyzed by qPCR once every month.

### Dissecting *F. virgata* Organs and Preparing for DNA Extraction

Las-infected *F. virgata* adults, reared on Las infected leaf discs, were dissected on glass slides under a stereomicroscope. For each sample of n = 25 mealybugs, a new razor blade was used to make an incision on the dorsal side of the insect and, with the aid of dissecting pins, the internal organs were isolated. The alimentary canal (Fig. S1), which is located mainly at the anterior of the abdomen, was washed twice with distilled water and then placed in a tube containing 150 µl of lysis buffer (0.1 M Tris-HCl, 0.05 M EDTA, 0.5 NaCl, 1% N-lauroylsarcosine, pH 8.0). The salivary glands, which are located anteriorly at the level of the stylets, were washed twice with distilled water and placed in a tube containing 150 µl of lysis buffer. The remaining body parts were placed in a tube containing 150 µl of lysis buffer. For each test, salivary glands and guts were collected from infected adults and non-infected adults as a control, and processed for DNA extraction and then qPCR assay.

### Insect and Leaf Disc DNA Extractions

Genomic DNA was extracted using the DNeasy Blood and Tissue Kit (QIAGEN, Valencia, CA). *Ferrisia virgata* and *D. citri* dissected tissues or whole bodies were homogenized in tubes containing glass beads using Fast Prep®-24 homogenizer (MP Bio., Solon, OH) at speed 4.0 m/s for 30 seconds. After homogenizing, 20 µl of proteinase K was added to each tube and the manufacturer’s protocol was followed. The DNA was eluted in 30 µl of nuclease-free water and analyzed by Nanodrop 1000 spectrophotometer (Thermo Scientific, Wilmington, DE) for DNA concentration and quality. qPCR reactions were run using TaqMan® assays and 16S rDNA-based primers and probe [27] to detect Las bacteria.

Leaf discs were collected and analyzed for Las bacterial titers. Each leaf disc was transferred to an autoclaved 2 ml screw tube containing two 4 mm silicone-carbide sharp particles and four 2.3 mm chrome-steel beads in 800 µL of extraction buffer (100 mM Tris-Base, 50 mM EDTA, 500 mM NaCl, 2.5% polyvinylpyrrolidone and 10 mM β-mercaptoethanol). They were homogenized by using a Fast Prep®-24 homogenizer at speed 6.0 m/s for 60 seconds and incubated at 65^o^C for 30 min after the addition of 80 µl 20% SDS. After adding one-third volume of 5 M potassium acetate, the tubes were incubated on ice for 5 min and centrifuged at maximum speed for 5 min to remove plant debris. The supernatants were removed and placed into a new tube and centrifuged for an additional 10 min at the same speed and 800 µL of supernatant was transferred to a new 1.5 ml tube containing two-third volume of cold isopropanol. The sample was then placed in a Genesee column (Genesee Scientific, San Diego, CA), centrifuged 1 min at 8000 rpm and washed twice with 70% ethanol. Samples were then eluted with 100 µl nuclease-free water, and then analyzed by the Nanodrop and adjusted to the concentration of 50 ng/µl DNA prior to qPCR analyses.

### qPCR Analyses

The TaqMan® qPCR assay was used to determine the presence of Las in mealybugs. Each PCR assay included negative and positive controls. TaqMan® real-time PCR amplifications were performed in a 7500 Real-Time PCR System (Applied Biosystems, Foster City, CA, U.S.A.) using primers HLBasf, HLBr and probe HLBp targeting the 16S rDNA of Las [28], [29] 15 µl of qPCR reaction mixtures contained 7.5 µl TaqMan® PCR master mix (Applied Biosystems), 250 nM each primer, 150 nM probe, and 100 ng template DNA. Real-time PCR data were analyzed by Applied Biosystems 7500 system SDS software version 1.2. The PCR program started with a denaturation step of 95°C for 10 min followed by 40 cycles of 95°C for 15 s and 60°C for 60 s.

### Analyses of Las Populations in Mealybugs and Psyllids

The Las populations in whole or dissected mealybugs (n = 50) and psyllids (n = 50) were studied by conventional PCR using primer pairs LJ799/858, LJ868/864 and LJ860/863 specific for Type A, B and D, respectively [24]. In brief, genomic DNA was extracted using the DNeasy Blood and Tissue Kit (QIAGEN, Valencia, CA). *Ferrisia virgata* and *D. citri* dissected tissues or whole bodies were homogenized in tubes containing glass beads using Fast Prep®-24 homogenizer (MP Bio., Solon, OH) at speed 4.0 m/s for 30 seconds. After mixing, 20 µl of proteinase K were added to each tube, and then followed the manufacturer’s protocol. The DNA was eluted in nuclease-free water and analyzed by Nanodrop for DNA concentration and quality. Leaf discs were collected and analyzed for Las bacterial titers. All the leaf discs or insect DNA samples were subjected to Las titer estimation by qPCR using 16S rDNA-based primers and probe [27] prior to typing analysis. Conventional PCR was performed on the T100 thermal cycler instrument (Biorad, Hercules, CA, USA) following the protocol as described previously [24].

## Results

### 100% Mealybugs Acquired Las Bacteria with a Simple Leaf Disc Bioassay

**Figure 1:**
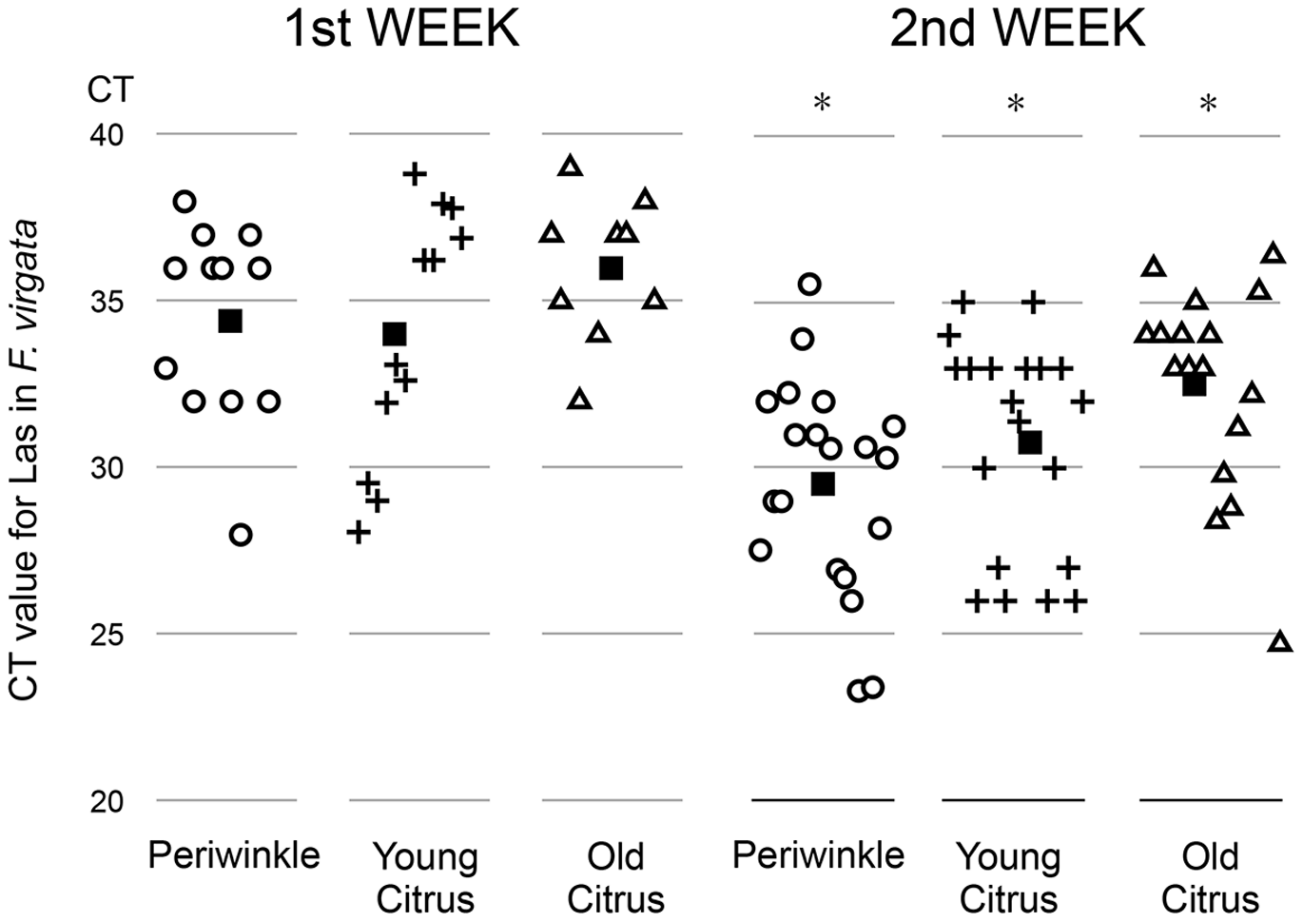
Ct values of single mealybug *(Ferrisia virgata)* after feeding for one and two weeks on ‘*Candidatus* Liberibacter asiaticus’ (Las)-infected periwinkle, young citrus and old citrus leaf discs. The black squares indicate the Ct means. The presence of Las was assessed using 16S rDNA-based primers and probe [27]. Asterisks indicate that the mean Ct value from the second week is significantly different from the first week’s mean Ct value. (Periwinkle (p<0.002), young citrus (p<0.013), and old citrus (p<0.0069) using Student’s t-test.).

By confining the feeding of mealybugs on leaf tissue with a high Las titer, the leaf disc bioassay increased the acquisition of Las by mealybugs compared to those reared on whole infected plants. After mealybugs fed on positive leaf discs for one or two weeks they were tested by qPCR using 16S rDNA-based primers and probe [27] (Fig. 1). Leaf disc plates were used for acquisition and transmission studies with periwinkle, old citrus and young citrus leaves.

The capacity of mealybugs to acquire Las from positive leaf discs was evaluated in two week-long experiments. Our results indicated that mealybugs were able to acquire Las bacteria from citrus and/or periwinkle after a one-week feeding period with an incremental increase in the Las titer after two weeks (Fig. 1). The percentage of Las-infected *F. virgata* was 100% in the first week when feeding on periwinkle and young citrus leaf discs and 75% when feeding on old citrus leaves. The average Ct values were 34.4, 34.0 or 36.1 using 16S rDNA-based primers and probe [27] for insects reared on periwinkle, young citrus or old citrus, respectively.

**Figure 2:**
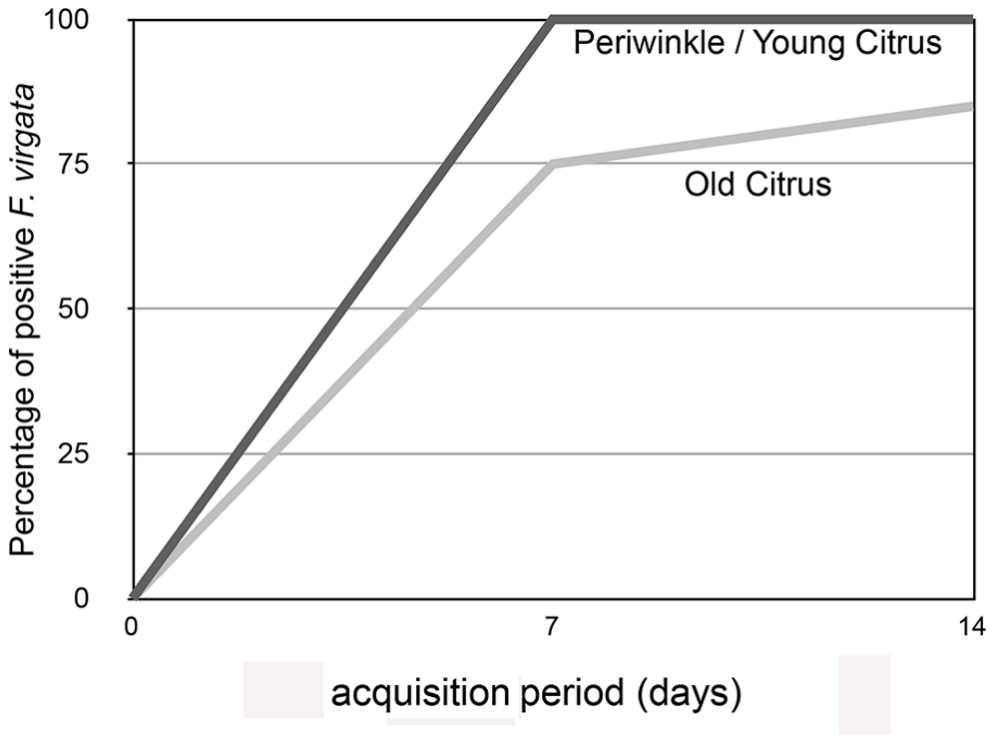
Average Ct values of *Ferrisia virgata* that fed for two weeks on ‘*Candidatus* Liberibacter asiaticus’ (Las) infected leaf discs and moved to non-infected leaf discs. For each sample day, means ±SE followed by different letters are significantly difference (P<0.005) using Student’s t-test.

The bacterial populations were estimated on the basis of three rRNA operons (containing the PCR target) per cell based on the established equation: Y = 13.3–0.299X [30]. The estimated Las population was 5.51×10^4^ Las cells per mealybug reared on periwinkle and young citrus, and 1.45×10^4^ Las cells per mealybug reared on old citrus (Fig. 2). The titer was positively correlated with the confinement time on the infected leaf discs, and after two weeks Ct values ranged from 23.1 to 36.1 (8.24×10^7^ to 1.07×10^4^ Las cells per mealybug). The average Ct value was 28.3, 33.2 or 34.3 for insects reared on periwinkle, young citrus and old citrus, respectively (Fig. 1B). The acquisition rate of Las after two weeks was 100% for mealybugs fed on either periwinkle or young citrus leaf discs, and 85% for mealybugs fed on old citrus leaf discs. All mealybugs collected from non-infected plants tested negative for Las bacteria.

In comparison, we also used whole infected periwinkle plants to determine Las acquisition by mealybugs. After 30 days on infected plants only 63% of the mealybugs collected from Las-infected periwinkle tested positive for Las bacteria (data not shown).

### Retention of Las Bacteria in the Mealybugs were Time and Host Plant-dependent

The retention of Las in mealybugs was assessed after rearing the insects for one to two weeks on infected periwinkle, young citrus, or old citrus leaf discs (Figure S1A). After one week of Las acquisition on leaf discs, mealybug nymphs tested positive for Las bacteria, but when the insects were transferred to healthy periwinkle or citrus leaf discs for an additional week, and collected for qPCR analysis, none of these insects tested positive for Las bacteria. However, when mealybugs were reared for two weeks on infected leaf discs and then moved to healthy leaf discs for an additional two weeks, they tested positive for the presence of the Las bacteria, indicating that the mealybug may acquires the Las bacteria during the first week, but only during the second weeks does the Las bacteria became systemic. The average Ct values were 30.4 (6.10×10^5^) for periwinkle, 35.6 (1.89×10^4^) for young citrus, and 36.1 (1.36×10^4^) for old citrus (Fig. 2). The proportion of Las positive adults decreased over time. Interestingly, the decrease of Las titers was more pronounced in mealybugs that fed on citrus leaf discs compared to periwinkle leaf discs. An additional analysis was conducted using a whole non-infected periwinkle plant. Mealybugs reared for two weeks on infected leaf discs were moved to non-infected whole periwinkle plants for four additional weeks (Fig. 2). The Ct values were recorded after two, three and four weeks. After two weeks on non-infected leaf discs, the Las average Ct value was 29.9 (7.63×10^5^ cells per mealybug). After two weeks on non-infected periwinkle plants, the average Ct value was 31.2 (3.12×10^5^ cells), after three weeks it was 31.1 (3.34×10^5^ cells), and after four weeks it was 30.7 (4.40×10^5^ cells). After four weeks, mealybugs were removed from the whole plants and the plants were subsequently tested every month for twelve months using Las-specific qPCR detection to check for the presence of Las bacteria. The results indicate all inoculated periwinkle plants were Las-negative.

### Low Number of Las Bacteria was Transmitted to the Leaf Discs of Host Plants

Individual Las positive mealybugs successfully inoculated periwinkle and citrus leaf discs after feeding for two weeks with resulting average Ct values of 36.5, 36.3 and 37.7 for periwinkle, young citrus and old citrus, respectively. The mean efficiency of inoculation was between 16 and 25% (Table 1). The transmission of Las bacteria implies that the HLB-causing bacteria circulate, infect, and/or replicate in various organs and tissues of the vector, including the alimentary canal and salivary glands, as reported by Ammar et al. [31]. These two organs are recognized as the most important transmission barriers of persistently and preoperatively transmitted plant pathogens, including viruses and bacteria. To transmit a plant disease, pathogens have two important barriers to traverse, the alimentary canal and salivary gland membranes [32], [15], [19], [16]. The presence of Las has been reported in the salivary glands and alimentary canal tissues of *D. citri* [31].

**Table 1:** Ct value of ‘*Candidatus* Liberibacter asiaticus’ (Las) in host plant leaf discs after inoculation with individual adult *Ferrisia virgate*.

Periwinkle leaf discs			Young citrus leaf discs			Old citrus leaf discs		
Las positive	Mean Ct value	SE	Las positive	Mean Ct value	SE	Las positive	Mean Ct value	SE
25%	36.5	0.43	20%	36.2	0.25	16%	37.7	0.8

Leaf discs were collected and processed for qPCR after being fed upon by Las-infected mealybugs for 2 weeks. Leaf discs without mealybug were used as control, which all tested Las negative.

The qPCR results of four tests conducted on dissected *F. virgata* organs indicated that Las was able to cross the alimentary canal and salivary gland membranes. Adult mealybugs were dissected under a stereomicroscope and tissue organs were collected for qPCR analysis (Figure S1B, C). Salivary glands, alimentary canals and the rest of the body were dissected from positive *F. virgata* adults. The average Ct value was 27.5 (3.98×10^6^ cells) from the gut, 37.5 (4.08×10^3^ cells) from the salivary glands and 32.1 (1.68×10^5^ cells) from the rest of the body. Salivary glands were Las positive in 30% of the cases with a Ct value ranging from 36.5 (8.12×10^3^ cells) to 37.8 (3.32×10^3^ cells) (Table 2a). The Las titer was significantly higher in the gut compared to the salivary glands and other body parts (P<0.001). An additional dissection analysis was done using infected mealybugs reared on whole non-infected periwinkle plants for four additional weeks. The average Ct values from the guts and the rest of the bodies were similar to those of the mealybugs grown for two weeks on infected leaf discs. However, the Las titer in the salivary gland tissue increased, with the average Ct value dropping from 37.5 (9.43×10^3^ cells) to 30.9 (4.08×10^5^ cells), indicating either movement of Las bacteria from the alimentary canal to the salivary gland or replication (Table 2b).

**Table 2:** Ct value of ‘Candidatus Liberibacter asiaticus’ (Las) in Ferrisia virgata organs based on qPCR for DNA from dissected adults and other body parts.

A								
Alimentary canal			Other body parts			Salivary glands		
Las positive	Mean Ct value	SE	Las positive	Mean Ct value	SE	Las positive	Mean Ct value	SE
100%	27.5a	0.75	100%	32.1b	0.56	30%	37.5c	0.64

**B**								
**Alimentary canal**			**Other body parts**			**Salivary glands**		
Las positive	Mean Ct value	SE	Las positive	Mean Ct value	Se	Las positive	Mean Ct value	SE
100%	27.5a	1.7	100%	33.6 b	1.5	50%	30.9 c	0.4

For each test healthy control adults were dissected and processed for qPCR. None were Las positive. Adults were collected after 2 (A) and 4 (B) weeks on infected leaf discs. Means followed by different letters are significantly different (P<0.001) using Student’s t-test.

Although Las infected mealybugs were able to transmit detectable level of Las to periwinkle and citrus leaf discs, none of the samples from the inoculated whole plants using a clip cage system tested positive for Las bacteria over the course of twelve months (data not shown).

### Different Las Bacterial Populations Existed in Infected Mealybugs and Psyllids

**Figure 3:**
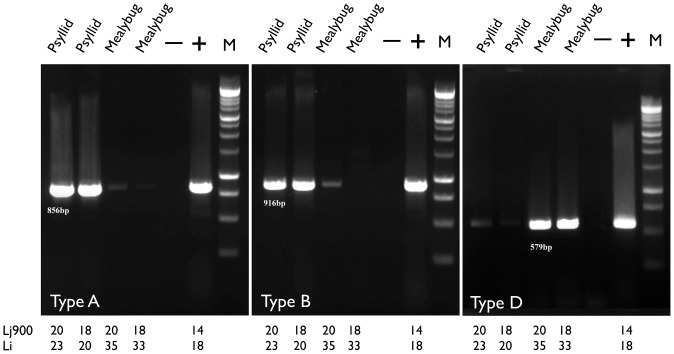
PCR amplification of the different ‘*Candidatus* Liberibacter asiaticus’ (Las) prophage variants [24]. Primers for Type A targets Las prophage FP1, Type B targets Las prophage FP2 and Type D targets the Las prophage iFP3. Using two sets of primers, Li and LJ900 [27], [29], targeting the Las 16S rRNA and prophage sequences, respectively, there was a fold-gap difference between the Li and LJ900 Ct values between psyllids and mealybugs.

In a previous study, nine types of Las variants were identified from Las infected plants and psyllids with four types being abundant (A, B, C, and D) and five types being rare (E, A1, A2, C1 and C2) [24]. Types A and B were located in the Las prophages, FP1 and FP2, respectively [33] and were confirmed to exist in Las-infected citrus, periwinkle and psyllid hosts. However, Type D was found in Las-infected plant hosts but not in psyllids. When infected mealybugs and psyllids were analyzed using molecular markers specific to the Las prophage/phage variants, Type A, B and D [24], different populations of phages/prophages were found in mealybugs compared to psyllids (Fig. 3). Interestingly, all mealybugs tested contained a high level of Type D and very low levels of Types A and B, contrary to psyllids in which Types A and B were the most abundant and Type D was absent (Fig. 3).

There was a small gap between the Ct values attained using the standard Li primers, which target the Las16S rRNA, and the LJ 900 primers designed to target the repeats of prophage/phage for any of the prophage populations in the psyllids [33]. However, the gap of the Ct values from two qPCR analyses using two different primer sets was noticeably bigger in the tested mealybugs (Fig. 3). This may indicate high levels of phage activity in the infected mealybugs. The prophage/phage populations were found to be different in psyllids and mealybugs, which may be related to successful or unsuccessful transmission of Las bacteria to the host plants.

## Discussion

HLB, one of the most important diseases of citrus, is primarily transmitted by insect vectors, especially the Asian citrus psyllid, *D. citri* [34], [35], [36]. The Las bacteria are able to infect various organ tissues of the vector, including the alimentary canal and salivary glands that are the most important barriers to successful transmission [34], [17], [11], [18]. In this study, we revealed that the striped mealybug can acquire Las bacteria and retain them stably in organs, including salivary glands, but was not able to transmit the bacteria to, and cause disease in, whole periwinkle or citrus plants (Fig. S1C).

Two different assay systems were utilized: a leaf disc bioassay [37], [26] and a whole plant assay. To understand the acquisition and transmission processes, we developed a bioassay for rearing mealybugs on leaf discs using water agar as a support instead of whole leaves. This system allowed us to control several critical aspects of the experiment: 1) the leaf surface area where the mealybug can acquire or transmit the bacteria was normalized; 2) the area where the mealybug can transmit the bacteria was reduced; and 3) the Las titer dilution that occurs on a whole leaf was reduced. Acquisition of the pathogen by mealybugs was 100% after 1 week on Las infected periwinkle leaf disc. Moreover the titer was positively correlated with the confinement time on the infected leaf discs, and after two weeks, the Ct value was up to 23.1 (8.24×10^7^cells). Previous transmission analysis with *D. citri* reared on *Citrus sinensis* indicated an acquisition rate of 60 to 100% for nymphs after 5 weeks and 40% for adults [38]. It is worth noting that there was a difference in Las inoculation between whole plants and leaf discs assays. There was a low level of detectable Las bacteria in inoculated leaf discs while the Las bacteria were below the threshold of detection in the inoculated plants. The reason why the Las bacteria were below the threshold of detection in the inoculated leaves of whole plants remains to be investigated.

Although striped mealybugs can acquire and retain Las, the different Las titers detected in mealybug organs indicate that both salivary gland and alimentary canal membranes constitute barriers to Las transmission in *F. virgate* [31], [16]. Overall, the Las titer in the salivary glands was lower than that of the alimentary canal and other body parts. Only 30% of the salivary glands tested positive with Ct values ranging from 36.5 (8.12×10^3^ cells) to 37.8 (3.32×10^3^ cells) (Table 2a), which explains the low rate of Las transmission. Alimentary canals had the highest titer indicating that they are the first site of infection in the vector as previously shown for persistently transmitted bacterial and viral pathogens [14], [18], [39]. Interestingly, additional weeks on non-infected periwinkle did not influence the Las titer in mealybugs.

The retention of Las declined over a two-weeks period when Las-positive *F. virgata* adults were moved to non-infected leaf discs, indicating that the bacteria did not replicate, or else the replication was at a very low rate in the mealybugs. Similar results have been shown in Las-infected adult *D. citri* [38]. However, retention of Las was relatively stable over four weeks when positive *F. virgata* adults were moved to whole non-infected periwinkle plants (Fig. 2). These results imply poor replication or persistence of Las bacteria occurs in the mealybug’s organs. Previous data indicate that the Las acquisition in *D. citri* increases with a longer acquisition access period on infected citrus plants from 0% at one week to 39% at five weeks [38]. Interestingly, the increase in Las acquisition by *F. virgata* on leaf discs was also correlated with an increase in the length of the acquisition access period on an infected leaf disc. Moreover confining the insects to Las-positive leaf discs allows mealybugs to reach an infection rate of 100% after only one week when mealybugs were feeding on infected periwinkle or young citrus leaf discs and 85% on old citrus leaf discs (Fig. 2). A single adult psyllid was able to transmit the Las pathogen after a one-day inoculation access period (IAP). However, the overall success ratio did not exceed 6.3% with a single inoculation. This did increase to 66% after a 30-day IAP [40]. Individual positive adult mealybugs successfully inoculated 16% to 25% of periwinkle and citrus leaf discs with Las after two weeks IAP (Table 1).

Using Las specific primers targeting the 16S rRNA gene and a repeat gene in the phage/prophage region, we analyzed the salivary glands of psyllids and mealybugs. The Ct values differed marginally between these two sets of primers in psyllids, but substantially in mealybugs (Fig. 3). More importantly, mealybugs were found to contain much higher titers of Type D when compared with psyllids (Fig. 3). In particular, psyllids contain Type A and B which correspond to prophages FP1 and FP2, respectively [41]. In contrast, mealybugs carried mostly the Type D Las populations with very low titers of Types A and B. These results indicate that the phage may be more active in the mealybug than in the psyllid and that the population types appear distinct. The Las bacteria are able to move from the gut to the salivary gland in both psyllids and mealybugs; however, only the psyllid can transmit the Las bacteria back into citrus and cause disease. We speculate that the phage iFP3, which contains Type D [24], may influence Las transmission efficiency by expressing important genes, or by reducing the Las titer in the salivary glands by killing the bacteria in the process of phage iFP3 replication. Further work is required to understand the interactions between the vector, the pathogen and the plant, and the characterization of the related genes in the prophages may be critical for elucidating the key component(s) associated with Las transmission.

In conclusion, F. virgta displays both similarities and differences compared with *D. citri* in terms of acquisition and/or transmission of Las bacteria. Results also confirm the complex of Las bacterial populations and their association with insect transmission and disease development. Different Las populations found in the two phloem-feeding insects may explain why the bacteria transmitted by striped mealybugs did not cause disease in host plants. Understanding the factors in Las prophage(s) affecting Las transmission by insect vectors may lead to new HLB management tactics.

## Supporting Information

Figure S1 **Leaf disc bioassay and gross anatomy of the alimentary canal and salivary glands of** ***Ferrisia virgata*** **observed in unstained preparations by stereomicroscopy.** A) Leaf disc bioassay to determine ‘*Candidatus* Liberibacter asiaticus’ (Las) acquisition and transmission in *F. virgata* insects. B) Gross anatomy of the mealybug’s alimentary canal; abbreviations: amg, anterior midgut; fc, filter chamber; mt, malpighian tubule; pmg, posterior midgut. C) Gross anatomy of the mealybug’s salivary glands; abbreviations: sg, salivary glands; asg, accessory salivary gland.
(TIF)
