# Correction: The Phloem-Sap Feeding Mealybug (*Ferrisia virgata*) Carries ‘*Candidatus* Liberibacter asiaticus’ Populations That Do Not Cause Disease in Host Plants

**DOI:** 10.1371/journal.pone.0092757

**Published:** 2014-03-14

**Authors:** The PLOS ONE Staff

Figure 2 is incorrect. The authors have provided a corrected version here.

**Figure 2:**
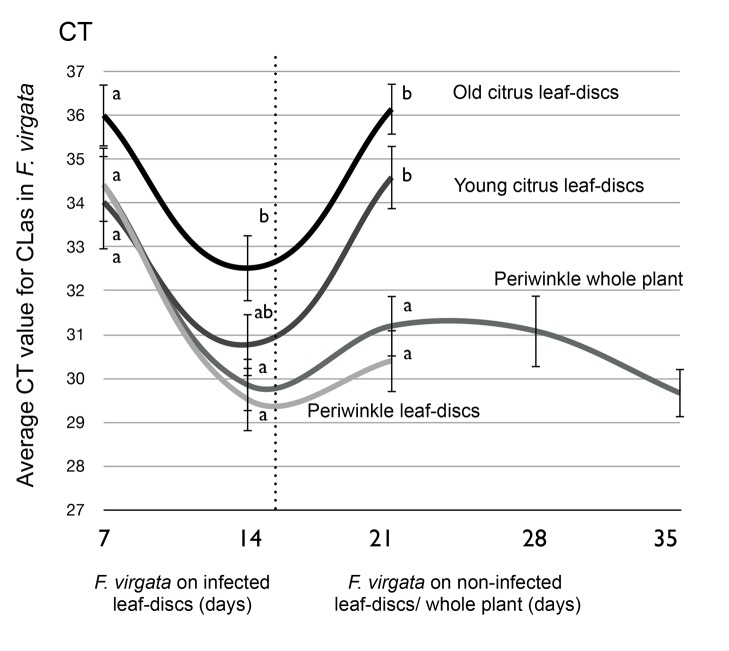
Average Ct values of *Ferrisia virgata* that fed for two weeks on ‘*Candidatus* Liberibacter asiaticus’ (Las) infected leaf discs and moved to non-infected leaf discs. For each sample day, means ±SE followed by different letters are significantly difference (P<0.005) using Student’s t-test.
