# Supplementary figures and images for: The Phloem-Sap Feeding Mealybug (Ferrisia virgata) Carries ‘Candidatus Liberibacter asiaticus’ Populations That Do Not Cause Disease in Host Plants
Source: PLoS One. 2014 Jan 20;9(1):e85503. doi: 10.1371/journal.pone.0085503 (PMC3896372; doi:10.1371/journal.pone.0085503)

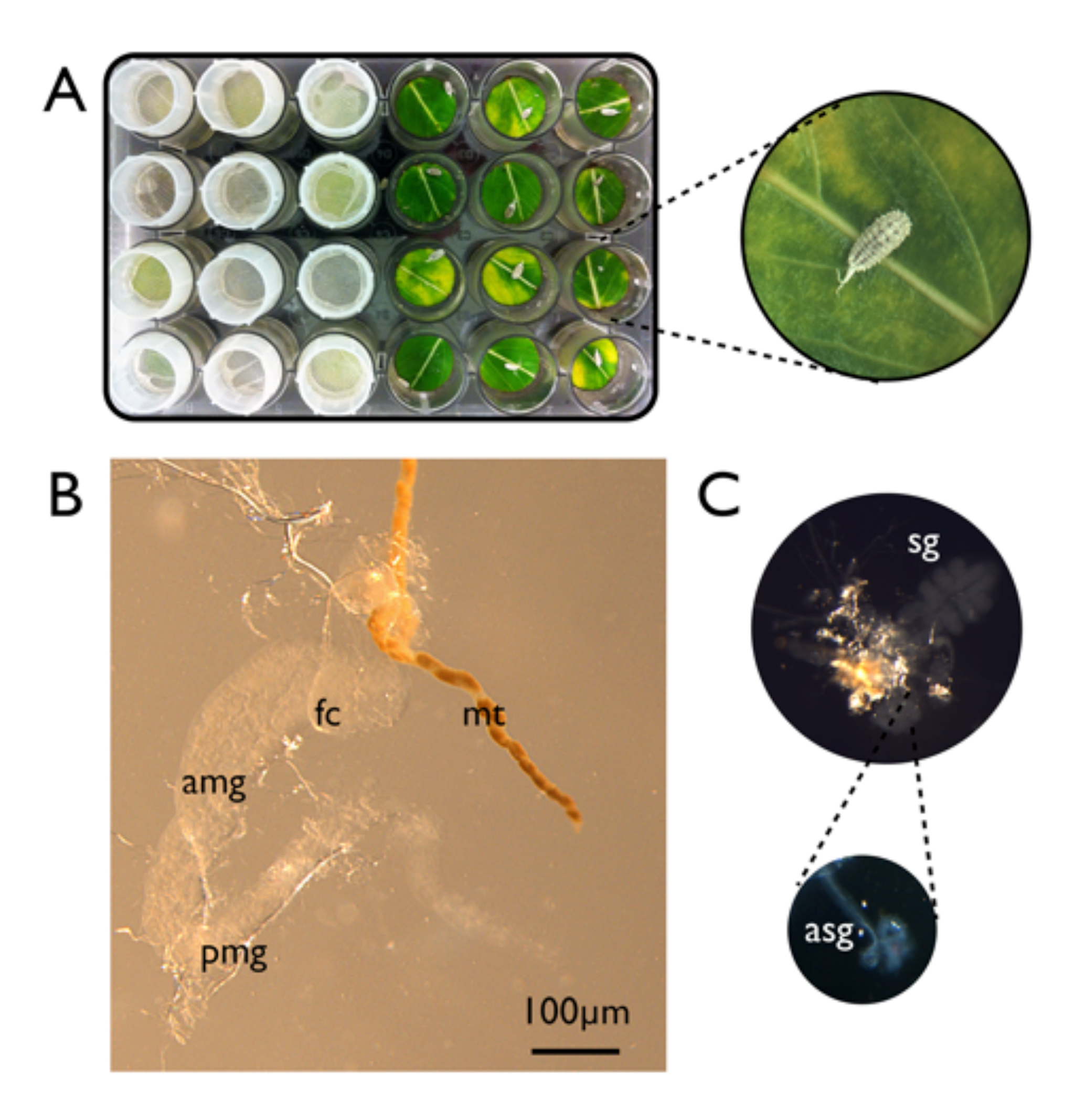

Supplement: Figure S1 — Leaf disc bioassay and gross anatomy of the alimentary canal and salivary glands of Ferrisia virgata observed in unstained preparations by stereomicroscopy. A) Leaf disc bioassay to determine ‘Candidatus Liberibacter asiaticus’ (Las) acquisition and transmission in F. virgata insects. B) Gross anatomy of the mealybug’s alimentary canal; abbreviations: amg, anterior midgut; fc, filter chamber; mt, malpighian tubule; pmg, posterior midgut. C) Gross anatomy of the mealybug’s salivary glands; abbreviations: sg, salivary glands; asg, accessory salivary gland. (TIF) [file pone.0085503.s001.tif]
